# RNA Vaccines against Infectious Diseases: Vital Progress with Room for Improvement

**DOI:** 10.3390/vaccines9111211

**Published:** 2021-10-20

**Authors:** Hana M. Abdelzaher, Asmaa S. Gabr, Basma M. Saleh, Rana M. Abdel Gawad, Ahmed A. Nour, Anwar Abdelanser

**Affiliations:** Institute of Global Public Health, School of Sciences and Engineering, The American University in Cairo, Cairo 11835, Egypt; hana-abdelzaher@aucegypt.edu (H.M.A.); asmaasaeed@aucegypt.edu (A.S.G.); basma_saleh@aucegypt.edu (B.M.S.); ranagawad@aucegypt.edu (R.M.A.G.); ahmed.adel.nour@aucegypt.edu (A.A.N.)

**Keywords:** mRNA vaccines, infectious diseases, challenges, clinical trials, SARS-CoV-2

## Abstract

mRNA vaccines have amassed a strong interest from scientists and nonscientists alike for their potential in treating cancer and curbing the spread of infectious diseases. Their success has been bolstered by the COVID-19 pandemic as mRNA vaccines for the SARS-CoV-2 virus showed unrivaled efficiency and success. The strategy relies on the delivery of an RNA transcript that carries the sequence of an antigenic molecule into the body’s cells where the antigen is manufactured. The lack of use of infectious pathogens and the fact that they are made of nucleic acids render these vaccines a favorable alternative to other vaccination modalities. However, mRNA vaccination still suffers from a great deal of hurdles starting from their safety, cellular delivery, uptake and response to their manufacturing, logistics and storage. In this review, we examine the premise of RNA vaccination starting from their conceptualization to their clinical applications. We also thoroughly discuss the advances in the field of RNA vaccination for infectious diseases. Finally, we discuss the challenges impeding their progress and shed light on potential areas of research in the field.

## 1. Introduction

RNA vaccines have become the frontline warriors in combating the COVID-19 pandemic. Although they caught the world’s attention with millions of people hearing about them for the first time, RNA vaccines are not unheard of. After 30 years of being nascent, interest has grown in these vaccines as a result of the COVID-19 pandemic due to a myriad of features, including their versatility—they could be tailored to fit the antigen of any infectious pathogen. In addition, their flexible and rapid production has been shown to fill the gap between a rapidly spreading disease pandemic and a direly needed vaccine for mass immunization [1]. RNA vaccines were still at the preclinical or clinical stages only until Pfizer/BioNTech (New York, New York; Mainz, Germany) and Moderna (Cambridge, Massachusetts) took their candidate mRNA vaccines: BNT162b2 and mRNA-1273 from bench to market. This was only after preliminary results of phase III clinical trials showed that these vaccines elicited immune responses with efficacy reaching up to 95% against COVID-19, indicating that this state-of-the-art technology could be promising and possessing potential for low-cost manufacturing with demonstrated safety, well-tolerability and immunogenicity [2,3]. Consequently, major investments were poured out by pharmaceutical companies and governments to drive this technology to the market. Prior to the emergence of SARS-CoV-2, RNA vaccines were also being developed to prevent infectious diseases such as influenza, zika, HIV, chikungunya, rabies and cytomegalovirus [4].

RNA or mRNA vaccines use single-stranded mRNA to produce an immune response. mRNA vaccine creation requires only the sequence code for the gene coding for a specific pathogen protein. The vaccine introduces synthetically produced mRNA into cells. The mRNA then causes the cells to synthesize the target protein that was supposed to be produced by the pathogen. Of note, mRNA vaccine functionality in antigen expression does not demand its entry to the host cell nucleus [5]. mRNA is responsible for the production of the desired protein only; it does not affect or change the genomic DNA sequence and is expressed temporarily, and then normal body mechanisms metabolize and eliminate it naturally accounting for its safety [6]. The produced protein from mRNA expression stimulates an immune response that is specifically tailored to destroy the corresponding pathogen. Thus, being reinforced with skyrocketing efficacy, safe administration and the ability to be customized for any antigen with minimal cost, mRNA vaccines represent a great alternative to conventional vaccines [7]. Currently, there are two types of RNA vaccination modalities. The first is conventional mRNA vaccines that encode desired antigen flanked by 5′ and 3′ untranslated regions (UTRs). They experience an uptake by cells via endocytosis and then released in the cell cytoplasm. A major advantage is their small size, making them simple to produce and minimizing un-needed immune responses [8]. The second type is self-amplifying (saRNA) mRNA vaccines, which are derived from a positive-stranded RNA virus genome. This mRNA vaccine encodes the desired antigen as well as replication machinery of the virus needed for the RNA amplification intracellularly resulting in high antigen expression levels [9].

In this review, we examine the history of RNA vaccination from vaccination to clinical applications as well as compare them to traditional vaccination modalities. We also highlight the different delivery methods of RNA vaccines. The strides in the development and implementation of RNA vaccination for infectious diseases are thoroughly discussed with a focus on SARS-CoV-2. Finally, we analyze the current challenges in RNA vaccination and shed light on potential areas of interest in the field.

## 2. RNA Vaccination from Conceptualization to Clinical Use

**Figure 1:**
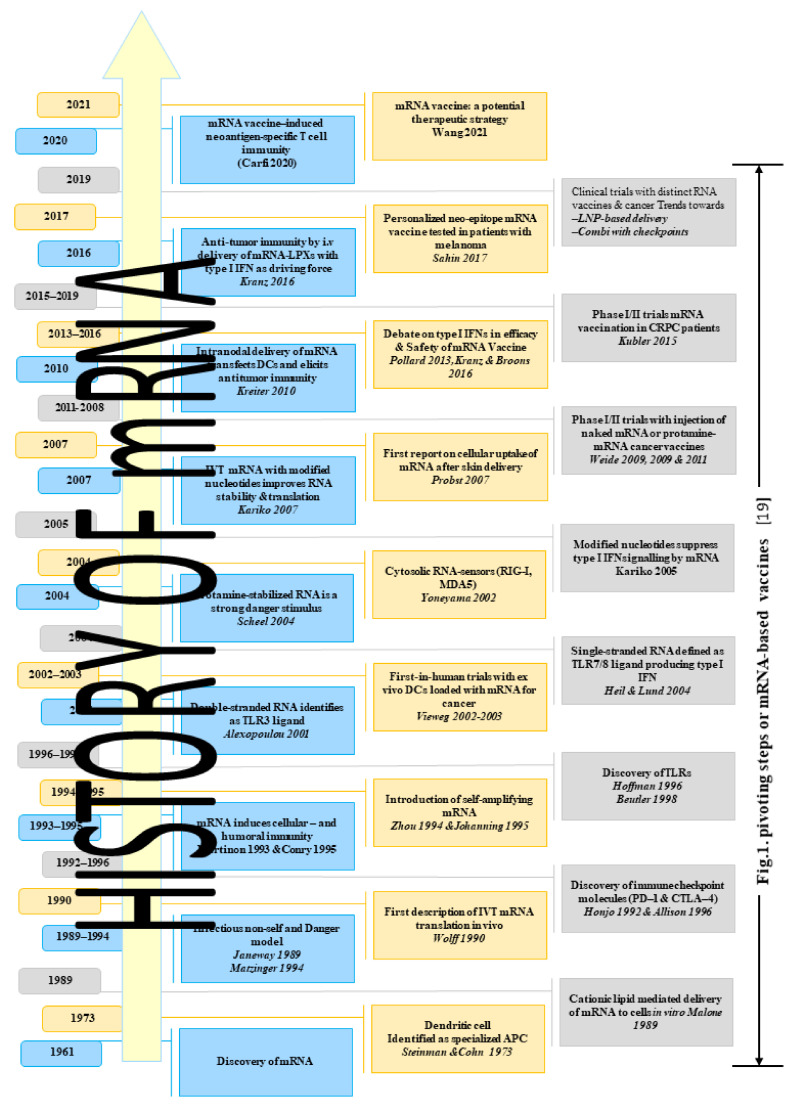
History of mRNA vaccination and their clinical applications.

In this section, we briefly discuss the history of mRNA vaccination and their clinical applications, as summarized in Figure 1. Messenger RNA (mRNA) was discovered within the same month of the splitting of the genetic code during the summer of 1961, in which two articles announcing the isolation of mRNA were published [10]. Later in the same month, another review paper was published in *Journal of Molecular Biology* in which mRNA was theoretically addressed, and its role in gene regulation was argued [10].

During the summer of 1989, Robert Malone and his team developed an efficient and reproducible technique using a synthetic cationic lipid to deliver mRNA to cells in vitro. They developed an efficient way to introduce mRNA into cells, and then the use of RNA transfection mediated by lipofectin (a liposome containing a cationic lipid) for efficient and reproducible RNA introduction and expression in tissue culture cells was then reported and published [11]. One year later (1990), Jon A. Wolff and his team separately injected RNA expression vectors containing genes for chloramphenicol acetyltransferase, luciferase and β-galactosidase into mouse skeletal muscle in vivo with no special delivery system. In all cases, protein expression was readily detected. This proved that injection of mRNA directly into the mouse skeletal muscle results in significant expression of reporter gene within the muscle in vivo [12]. Further development was achieved by Martinon and his team in 1993, who proved that mRNA induces cellular immunity. They induced anti-influenza cytotoxic T lymphocytes (CTL) in vivo by immunizing mice with liposomes containing mRNA encoding the influenza virus nucleoprotein (NP); with this mRNA liposome, virus-specific CTL responses could be elicited in mice [13].

In 1995, mRNA transcripts were constructed encoding luciferase and human carcinoembryonic antigen (CEA), and the CEA expression was directed in mouse fibroblasts in vitro following liposome-mediated transfection [14]. Self-cloning mRNA was concluded to be quite useful as a nucleic acid vaccine in 1994 [15]. This experiment was conducted using Semliki Forest virus (SFV) to express the nucleoprotein of the influenza virus in mice. In another study conducted by F. W. Johanning, the observation was that self-replicating mRNA was capable of directing elevated levels of reporter gene expression in myocytes compared to nonreplicative mRNA species [16].

In 2004, Eli Gilboa and Johannes Vieweg described the use of mRNA-encoded tumor antigens when loaded onto dendritic cells (DCs), and the result of their comparative studies suggested that mRNA transfection outweighs other antigen-loading techniques in generating immunopotent DCs [17]. Furthermore, in 2007, in vitro-transcribed mRNA was incorporated with naturally modified nucleotides into transcripts, the effect of this on the biological properties of mRNA was investigated. The results were that mRNAs containing pseudouridines have a higher translational capacity than that of unmodified mRNAs [18]. During the same year, the first cellular uptake of mRNA after skin delivery was reported, and a study was conducted by injecting naked mRNA in the skin, which resulted in the mRNA local uptake and expression by different cell types at the site of injection, and the protein translated from this was detected after just a few days [19].

From 2009 through 2011, Benjamin Weide et al. conducted phase I and II trials with vaccination of protamine-encapsulated mRNA in 21 metastatic melanoma patients. They concluded that direct injection of protamine-protected mRNA is feasible, safe and capable of promoting antitumor immunity [20]. The first report of strong systemic antigen-specific Th1-type immunity and cancer cure achieved with naked antigen-encoding RNA in preclinical animal models was in (2010) by Sebastian Kreiter [21]. A first in-man phase I and IIa study (self-adjuvanted mRNA vaccination in advanced prostate cancer patients) was conducted in (2015) by Hubert Kubler and his team on 44 advanced prostate cancer patients at 12 centers in Germany and Italy, and the result was 91% were evaluable for (prostate-specific antigen) PSA response [22]. In 2016, Lena Katnz proved that systemic RNA delivery to dendritic cells uses antiviral defense for cancer immunotherapy, and they also demonstrated how DCs can be effectively targeted in vivo using intravenously administrated RNA-lipoplexes (RNA-LPX) and found the strategy to be highly successful [23].

In 2017, Sahin and Ozlem conducted the clinical trials of personalized cancer vaccines, which have proven the feasibility, safety and immunotherapeutic activity of targeting individual tumor mutation signatures [24]. In 2020, another study was published in the *Journal of Clinical Investigation* in which the authors concatenated (validated, defined neoantigen and predicted neoepitopes and mutations of driver genes) into a single mRNA construct and used it to vaccinate patients with metastatic gastrointestinal cancer. The vaccine was found safe and elicited mutation-specific T-cell responses against predicted neoepitopes that were not detected before vaccination [25].

## 3. Mechanism of Action for mRNA Vaccines

RNA vaccines use the natural body immunity by directing the expression of antigen (coded on mRNA) in the host cell. Modifying RNA sequence in vaccines increases mRNA expression in the host cell and reduces the natural sensing of the host immune system [6]. The natural or engineered sequence of mRNA directs the antigen to the desired cellular location. mRNA vaccines use the host machinery translating mRNA into its related antigen resulting in a pseudoinfection similar to that caused by the intended virus through the release of cellular and humoral immune response [9]. Mutations in 3′ and 5′ UTRs in mRNA affect its translation and protect mRNA degradation by enzymes [26]. mRNA purity is a vital aspect affecting vaccine stability, the amount of protein production through the translation process and mRNA degradation [27]. There are two receptor families intracellularly that sense mRNA. Toll-like receptors (TLR-3,7,8,9) are present in the endosomal compartment in immune surveillance cells (DCs, monocytes and macrophages). The second family of receptors is known as pattern-recognition receptors (PRR) including RIG-1, MDA-5, LGP-2. Each of these receptor families recognizes different types of mRNA eliciting an immune response for the coded antigen [27].

mRNA is engulfed by both immune and nonimmune cells. For the immune cells, endosomal TLR7 and TLR8 sense mRNA and are triggered, leading to mRNA presentation in the endosomes followed by their release in cell cytoplasm where the mRNA-coded antigen is expressed. For the nonimmune cells, mRNA is recognized by cytoplasmic sensors (RIG-I and MDA5) inducing IFN expression leading to cytokines and chemokines production. Moreover, nonimmune cell death due to the high amount of protein expression is followed by APC uptake of the protein once it is released. The protein is then presented to CD4+ T cells. Therefore, nonimmune cells can activate innate immunity at the site of injection in addition to inducing humoral immunity through activating CD4+ T-cell via antigen presentation [28].

Moreover, mRNA vaccines activate the pattern-recognition receptor (PRR) and initiate immune response via production of chemokines and cytokines such as interleukin-12 and tumor necrosis factor at the site of injection. The chemokines and cytokines act as immunostimulatory moieties and activate lymph nodes through B cell proliferation and granulocyte recruitment. This is essential for inducing an effective immune response against the encoded antigen [28,29]. For lipid nanoparticle nonreplicating mRNA vaccines, they cause strong activation of the innate immunity. mRNA is taken up by cells around the injection site and then expressed inside these cells (APCs, neutrophils and nonleukocytic cells). This is followed by antigen-specific CD4+ T cells priming in the lymph nodes. mRNA vaccines also induce the production of type I interferon. The expressed antigenic proteins are processed to antigenic peptides and presented on major histocompatibility complex MHC class I and II in addition to costimulation of CD8+ and CD4+ T cells. B cells then recognize the antigen presented and produce antibodies against this antigen [8]. 

As for self-amplifying mRNA, it mimics the replication of a positive single-stranded mRNA virus, resulting in an increase in the duration and level of immunity expression against the coded antigen. A major advantage for this type of mRNA vaccine is that a small amount injected results in long duration of action and increased antigen production, resulting in a higher immune response and thus better host protection. Moreover, it can encode for several antigens in the same replicon, thus improving vaccine potency [9]. Following the delivery of the purified RNA into the host cell, it is extensively translated and amplified via RNA polymerase [27]. A major challenge for self-amplifying mRNA vaccines is the difficulty in scaling up production due to the long sequence of designed RNA [5].

Furthermore, mRNA vaccines have therapeutic use as well. For example, cancer mRNA vaccines code for antigen expression of the associated tumor or growth factor resulting in stimulation of cellular immunity against cancer cells causing their inhibition or clearance [26,27,30]. Developing personalized vaccines is being proposed since some cancer mutations are specific to an individual. mRNA vaccines have the flexibility to code for multiple antigens with same backbone and are easily manufactured [26]. A promising approach is designing mRNA vaccines with self-adjuvanticity that can improve their performance. Examples such as granulocyte-macrophage colony-stimulating factor (GM-CSF) mRNA enhanced cytotoxic T lymphocytes (CTL) activity and durability, depending on the dose, improving memory cells [31].

## 4. Methods of Delivery for mRNA Vaccines

The mRNA vaccine delivery system can affect the antigen protein expression quantity and quality, thus affecting the stimulation of the host immunity [9]. Cells uptake the mRNA via endocytosis and then attach and fuse with cell membrane electrostatically via nonbilayer lipid phase [26]. mRNA needs to be delivered to the host cell cytoplasm through plasma membrane for antigen expression eliciting a specific immune response [31,32]. Importantly, mRNA delivery systems affect the stability of mRNA vaccines and prevent its degradation, degree of immune response in terms of protein expression level and production of antigen-specific T and B cells, cytokines, interferons and level of neutralizing antibody titers [27]. Several delivery systems have been used to enhance mRNA vaccine penetration through the host cell lipid membrane.

First, some mRNA vaccines are delivered via protamine complexes inducing Th1 T cells. Antigen expression via mutated mRNA sequence highly depends on the protamine and mRNA ratio [26]. Naked mRNA combined with protamine causes strong antigen expression, while protamine part is a potent immunostimulatory [31]. Second, viral vectors for mRNA vaccine delivery showed safety problems with immunocompromised patients due to increased viral replication and difficulty in large scale production. Therefore, nonviral vectors are preferred in mRNA vaccine delivery due to their easy production and low immunogenicity in addition to protecting mRNA from enzymatic degradation and aiding its delivery to the targeted cell membrane [33]. Third, another delivery system for mRNA vaccines is nanosilica. It encloses the mRNA effectively and protects it from enzymatic degradation along its movement in the cell. Within the cell after endocytosis uptake, RNA is released in the cytosol to produce the targeted protein which elicits an immune response, producing humoral response (antibodies) and cellular responses (T cells) [34]. Furthermore, the proposed lipid-based formulations (LBF) for mRNA delivery are lipopolyplexes, lipoplexes, lipid emulsions and lipid nanoparticles. Moreover, some LBFs have pH-sensitive molecules such as fusiogenic or histidiylated lipids or protonable polymers to help mRNA escape endosome degradation [35].

However, the mostly favored delivery system is the lipid nanoparticle (LNP) [36] which is made up of four units: ionizable cationic lipid, polyethylene glycol linked to a lipid, cholesterol and natural phospholipids. LNP is a highly favorable delivery system, since it protects the mRNA from degradation, its tunable physiochemical properties and ability to produce a strong immune response with high antibody titers and enhanced T and B cells immunity. It also helps mRNA expression in the cell cytoplasm for translation [8,32]. Lipid formulation strategies are summarized in Table 1.

The possibility of adding targeting molecules to the LNP and LBP (such as glycomimetics or carbohydrates: glycotargeting) helps vaccine direction to specific cells or tissues, thus enhancing mRNA uptake by the targeted immune cell type, increasing immune response against the required antigen [8,30]. Composition of lipid nanoparticles is highlighted in Table 2. The mode of administration of the vaccine whether intradermal (ID), intramuscular (IM) or subcutaneous (SC) affects the duration and intensity of antigen expression, in addition to affecting immune cell activation in terms of the type of cells activated and the intensity of activation [9,26]. Especially during outbreaks, it is advisable to use a mode of administration that is reliable and easy for the medical team [8]. Depending on the intended use of mRNA vaccines, whether for therapy or prophylaxis, the route of administration is selected based on this. ID, IM and SC are mostly used in vaccinations, while intravenous and intraperitoneal are used mainly in therapy [26,27].

Several methods are used to enhance mRNA stability, delivery and the production of proteins. Methods include altered nucleosides and nanoparticles technology for delivery which stabilizes mRNA, improves its uptake by the host cell and enhances its bioavailability [5]. Short mRNA depending on robust induced silencing complex of RNA can be modified while maintaining its potency. Meanwhile, long mRNA sequences benefit from natural modification by having substitutes such as pseudouridine or 5-methylcytidine, as they need to be effectively translated by ribosomes [37].

## 5. RNA Vaccines vs. Traditional Vaccination Modalities

A vaccine typically consists of the antigen to which an immune response is desired in addition to other substances such as adjuvants, preservatives, and stabilizers. The antigen is the infectious agent that has been destroyed or damaged, rendering it harmless, and its introduction allows the human body to recognize and battle the illness if reinfected [38]. Vaccines are classified according to the antigen used in their development to whole-pathogen vaccines, subunit vaccines, nucleic acid vaccines and viral vectors or viruses such as particles. Their formulas have an impact on how they are used, stored and administered [39]. Whole-pathogen vaccines are traditional vaccines utilizing the whole organism to generate a live-attenuated or inactivated/killed form of the pathogen for the purpose of immunization [40,41].

The earliest used type of whole-pathogen vaccination strategies is live-attenuated viral vaccines. This method uses live pathogens and weakens them through different techniques. One of the most common methods is to cultivate the viruses in foreign hosts, such as animal cell cultures, where they reproduce poorly. Additional molecular strategies such as viral gene mutation or deletion, or codon deoptimization, may be used [42]. The second type is killed or inactivated vaccines. Here, the pathogen is killed by chemical or physical means, for example, by formaldehyde, formalin, radiation or heat. Inactivated vaccines are much safer and more stable than attenuated ones [42].

One of the most significant advances in the field of vaccination has been the ability to build an infectious clone with a complete viral genome sequence on a bacterial plasmid. In order to engineer genetically modified viruses, the viral genes on the contagious clone can be quickly manipulated and transfected into susceptible cells. For the desired result, a chimeric virus shuffled with different viral genomes can be created using this technique [43].

Subunit vaccines are yet another category of vaccines; they are produced using synthetic peptides or recombinant proteins. Unlike inactivated or live-attenuated virus vaccines and other viral vectored vaccines, subunit vaccines include only unique viral antigenic fragments and no infectious virus elements, mitigating the issues of incomplete inactivation, virulence regeneration or pre-existing immunity. Subunit vaccines are relatively healthy and do not elicit potentially adverse immune reactions, making them attractive vaccine candidates. Furthermore, subunit vaccines with improved immunogenicity and/or efficacy can target particular, well-defined neutralizing epitopes [44]. Virus-like particle (VLP) vaccines investigate the immunogenicity and stability of empty virus particles with many copies of the same antigen on the surface. They are engineered to replicate the virus structure, eliciting robust immune responses against the antigens displayed on their surface; they have higher safety profiles due to the absence of the pathogen’s genetic content [45].

Unlike conventional vaccines, gene-dependent vaccine platforms based on viral vectors, DNA and RNA have shown positive outcomes in terms of both humoral and cell-mediated immune responses. They depend on host cells to generate the target protein vaccine antigen, rather than the antigen being purified and administered directly. Nucleic acid vaccine ingredients are much cheaper to produce than purified antigens and have the advantage of being able to be produced quickly. There were no approved nucleic acid platforms for infectious diseases prior to the SARS-CoV-2 vaccines, yet for decades, scientists have studied and worked with mRNA vaccines as they can be created in a laboratory using readily available materials. This means that the procedure can be streamlined and scaled up, allowing for quicker vaccine production than conventional approaches, and so, the successful use of this tool aided the accelerated progression of the SARS-CoV-2 vaccines [46,47]. These various types of vaccines have the same role of providing immunity to the host against specific antigen/antigens, but they differ in their formulation and accordingly in the nature of immunity triggered, safety, efficacy, rapidness and how they are used/administered.

The advantage of mRNA vaccines, like all vaccines, is that vaccinated individuals receive immunity without ever having to face the severe effects of administering the pathogen itself, either attenuated or even killed. mRNA vaccines have shown a number of distinct benefits over traditional vaccines. To begin with, mRNA can ideally fulfill all genetic material criteria for encoding and expressing all types of proteins. Vaccine development production can be improved by altering the mRNA series, which is a more convenient method than other types of vaccine modification; therefore, it is easily produced with short production time and also easily modified [7,47].

mRNA vaccination is a highly efficient method of giving active immunity against the infectious agent. It produces a high amount of protein, resulting in highly efficient and long-lasting immunity [27]. In contrast to DNA-based vaccines, mRNA vaccines are much more potent in expressing target proteins due to their capability to express these proteins directly in the cytoplasm rather than the nucleus. Furthermore, since the chemical structure of the mRNA sequence differs from that of DNA, mRNA has a lower chance of integrating into the host DNA genome and inducing a smaller immune rejection response, such that this technology proved to be tolerable and to have a high safety profile [27,31,48].

While there is no real-world experience with immunodeficient patients, certain possible benefits of RNA-based vaccines for this particular population should be considered. Recent research has shown that mRNA vaccines outperform other vaccines such as live-attenuated, protein subunits, inactivated, and DNA vaccines because mRNA is a noninfectious, nonintegrating vector/instruct vector [49].

## 6. RNA Vaccines for Infectious Diseases: Where Do RNA Vaccines Stand in Clinical Trials?

In this section, we review the progress of RNA vaccines for infectious diseases in clinical trials. A summary of this information can be found in Table 3 and Table 4.

### 6.1. SARS-CoV-2

RNA vaccines against SARS-CoV-2 are now among the most extensively studied vaccines ever since, in December 2020, Moderna’s mRNA-1273 and Pfizer/ BioNTech’s BNT162b2 were approved for emergency use by the U.S. Food and Drug Administration (FDA) and European Medicines Agency (EMA). On the other hand, there are still 10 other vaccines against COVID-19 in ongoing clinical trials, and 24 other COVID-19 vaccines are still in preclinical development to date [50]. A detailed review of mRNA vaccines for COVID-19 is found in Table 4.

**Table 4:** Clinical trials with mRNA vaccines against viral diseases [51].

Infectious Disease Type/Virus Type	NCT Number	Drug Administration	Phase	Status
SARS-CoV-2	NCT04523571	BNT162b1 + placebo	I	Recruiting
	NCT04449276	CVnCoV Vaccine + placebo	I	Recruiting
	NCT04470427	mRNA-1273 + placebo	III	Recruiting
	NCT04368728	BNT162b1 + BNT162b2	I/II/III	Recruiting
	NCT04515147	CVnCoV	IIA	Not yet recruiting
	NCT04283461	mRNA-1273	I	Active, not recruiting
	NCT04405076	mRNA-1273 + placebo	IIA	Active, not recruiting
Rabies	NCT02241135	CV7201 mRNA encoding the rabies virus glycoprotein	I	Completed
	NCT03713086	Rabipur^®®^	I	Active, not recruiting
HIV-1 Infection	NCT00833781	mRNA-transfected autologous DCs+/− autologous DCs with no mRNA transfection	I/II	Completed
	NCT02413645	TriMix mRNA+/−HIV mRNA	I	Completed
	NCT02888756	iHIVARNA-01 + TriMix+/−Placebo	IIA	Terminated
Zika Virus	NCT03014089	mRNA-1325 + placebo	I	Completed
	NCT04064905	mRNA-1893 + placebo	I	Active, not recruiting
Tuberculosis	NCT01669096	GSK 692342	II	Completed
Human Metapneumovirus and Human Parainfluenza Infection	NCT03392389	mRNA-1653 + placebo	I	Completed
	NCT04144348	mRNA-1653 + placebo	Ib	Recruiting
Ebola Virus Disease	NCT02485912	Two separate RNAs encoding two Zaire strain ebola glycoproteins, respectively	I	Completed
Influenza	NCT03076385	VAL-506440 + placebo	I	Completed
Respiratory Syncytial Virus	NCT04528719	mRNA-1345 + placebo	I	Not yet recruiting
Cytomegalovirus Infection	NCT03382405	mRNA-1647, mRNA-1443	I	Active, not recruiting
	NCT04232280	mRNA-1647 + placebo	II	Active, not recruiting

Moderna’s mRNA-1273 encodes the spike protein of SARS-CoV-2 encapsulated in a novel lipid nanoparticle (LNP). This versatile mRNA vaccine entered its phase I clinical trial on healthy participants in less than 10 weeks from publishing the first genome sequence of SARS-CoV-2, which was considered unprecedented in the pharmaceutical industry history. In November 2020, the primary results of phase III demonstrated that seroconversion took place in all study participants, and immunogenicity response lasted for at least 119 days after the first vaccination and was greatly influenced by the administered dose in addition to 94.5% efficacy in preventing the SARS-CoV-2 infection for severe cases of the disease that also showed no significant safety concerns [52]. On the other hand, systemic adverse events (AEs) were more common with higher doses of mRNA-1273 (reported in 33% participants) [53]. In addition, Moderna expanded its vaccine candidates to dispute the SARS-CoV-2 circulating variants by developing mRNA-1273.351 vaccine, which is also an LNP-encapsulated mRNA-based vaccine but encodes for the full-length Spike protein of the SARS-CoV-2 B.1.351 variant. In March 2021, a phase I trial for mRNA-1273.351 vaccine was initiated to assess the safety, well-tolerability and immunogenicity of mRNA-1273.351 vaccine in previously vaccinated individuals and naïve ones [54]. 

The mRNA BNT162b2 (Pfizer/BioNTech) vaccine is formulated in a versatile lipid particles system that elicits immunogenicity against SARS-CoV-2 spike protein. The vaccines showed promising results for early protection when 52% efficacy was observed after the first dose that was followed by 95% efficacy in preventing the SARS-CoV-2 mild to serious cases of infection after the second dose, leading this candidate vaccine to be chosen by the US government for emergency use authorization (EUA) immediately after announcing the conclusion of their phase III trials [3]. Adverse events for this vaccine ranged between injection site reactions, fatigue, headaches, and fevers (reported in 27% of patients) [3]. A recent study evaluated the effectiveness of BNT162b2 mRNA vaccine in a nationwide mass vaccination setting showed high effectiveness of the BNT162b2 vaccine for preventing symptomatic COVID-19 in a noncontrolled setting in addition to high protection against the more serious outcomes: hospitalization, severe illness and death [55]. A long-term phase IV study was implemented with larger sections of the population in February 2021 to study the effectiveness of mRNA-1273 and BNT162b2 mRNA vaccines, durability as well as safety of citizens being vaccinated with one of these SARS-CoV-2 vaccines in general population.

Another mRNA vaccine (CVnCoV) was developed by Curevac (Tübingen, Germany) using synthetic strands of mRNA without chemical modifications formulated as mRNA in LNP and encoding the full-length SARS-CoV-2 spike protein. Data obtained from assessing CVnCoV on various animal models showed that this vaccine elicits immune response comparable to that manifested in the convalescent sera of infected persons in addition to inducing specific T-cell responses [56]. Phase I and phase II trials for CVnCoV were launched in adults aged 18–60 years to evaluate the safety, reactogenicity profile and humoral immune response after 1 and 2 dose administrations of CVnCoV at different dose levels [57]. The vaccine is still under phase III trial which was initiated in December 2020 to assess the safety, immunogenicity and effectiveness against COVID-19. Subjects are enrolled in multiple European and Latin American sites and follow a two-dose schedule of 28 days apart [58].

ARCT-021 is an saRNA vaccine against SARS-CoV-2 developed by Arcturus Therapeutics (San Diego, CA, USA). Unlike the previously mentioned mRNA vaccines, this candidate vaccine utilizes a self-transcribing and replicating RNA (STARR) technology and is delivered in a lipid-enabled and unlocked nucleic acid modified RNA (LUNAR) system. This modality led ARCT-021 to not rely on any viral vectors or adjuvants. Preclinical studies conducted on animal models showed that a 2 µg dose of this candidate vaccine has the ability to increase neutralizing antibodies after 60 days of administration due to protein expression sustainability [59]. At a cellular immune response level, it induced robust CD8+ T-cell induction and a Th1-biased T-helper [59]. Consequently, phase I/II study for this candidate vaccine was initiated on July 2020, to investigate the safety and immunogenicity of ARCT-021 in healthy participants. This study was designed in two separate phases, including Phase I where safety and immunogenicity of escalating doses as a single injection were investigated in 21–55 years of age healthy volunteers then administration of two doses in phase II is planned to further evaluate ARCT-021 efficacy in younger adults (21–55 years) and elders (56–80 years).

MRT5500 developed by Sanofi and Translate Bio in a preclinical study demonstrated the ability to elicit neutralizing antibodies using a two-dose schedule administered 3 weeks apart [60]. Despite this, Sanofi phase I/II trial to evaluate the safety, tolerability and immunogenicity of MRT5500 is still ongoing. Clinical trial participants are expected to receive one dose of MRT5500 or two doses 21 days apart [61]. Sanofi and Translate Bio announced that they are working thoroughly to overcome the extreme low temperature paradox for their candidate vaccine by improving its temperature stability to reach a −20 °C storage temperature for late-stage clinical trials. Furthermore, the possibility to maintain this vaccine stable at routine refrigerator temperatures (2–8°C) was identified [62].

LNP-nCoVsaRNA developed by Imperial College of London is another saRNA vaccine candidate that is derived from an alphavirus genome and encodes the alphaviral replicase and SARS-CoV-2 prefusion stabilized spike protein. A preclinical study of this vaccine demonstrated that administration of two doses of this vaccine induced higher neutralizing antibody titers in compare with convalescent sera of recovered COVID-19 patients in addition stimulating IFN-γ immune responses [63]. The vaccine is in phase I/II trials with different dose levels of the vaccine being evaluated on study subjects of 18–45 years of age [64]. Imperial College of London team is planning to implement an inhaled dose-ranging trial for orally inhaled vaccine that could potentially accelerate the development of effective vaccines against COVID-19 by exploring additional delivery methods and targets that could induce a localized, and potentially more specialized, immune response [65].

The Academy of Military Medical Sciences, Suzhou Abogen Biosciences and Walvax Biotechnology developed an LNP-encapsulated nucleoside-modified mRNA encoding the receptor-binding domain (RBD) portion of the SARS-CoV-2 spike protein, ARCoV. A preclinical study on this vaccine successfully showed that the administration of two doses of ARCoV resulted in complete protection in mice against the challenge of a SARS-CoV-2 mouse-adapted strain. In nonhuman primates, robust levels of neutralizing antibodies were elicited against SARS-CoV-2 in addition to Th1-biased cellular response [66]. As a result, phase I and phase II clinical trials were carried out to explore the safety, tolerability and immunogenicity of different doses to the RBD of S protein in population aged 18–59 years and 60 years and above.

Researchers at Thailand’s Chulalongkorn University have been developing an mRNA potential vaccine for the coronavirus, the ChulaCov19 vaccine. Pre-clinical results of this vaccine showed that mice received the full dose of 2 injections of the ChulaCov19 vaccine, 3 weeks apart then got infected with COVID-19 were protected from the virus before its entry to the bloodstream. In addition, the virus count in the nose and lungs was reduced by 10,000,000 times [67]. They also announced that ChulaCov19 can be stored at a normal refrigerator’s temperature of 2–8 °C for at least one month [67]. On September 2020, a phase I/II trial was registered to test the ChulaCov19 vaccine in humans. The first phase of the study was planned to determine the safety, tolerability and reactivity to ChulaCov19 vaccine administered at various doses among healthy adults and the elderlies. As a result, phase II proceeds to explore the vaccine ability to activate the immune system and elicit cellular response among healthy adults and elderlies or not [68].

Providence therapeutics is still recruiting for its phase I trial for its PTX-COVID19-B mRNA vaccine. This study aims to evaluate the safety, tolerability and immunogenicity of PTX-COVID19-B vaccine in healthy seronegative adults aged 18–64. Various doses will be administered to the study subjects with 28 days. GSK is currently also evaluating a CoV-2 self-amplifying mRNA vaccine encoding the SARS-CoV-2 spike protein formulated with LNP in healthy adults 18–50 years of age at four different doses on a 1 month dosing period; this study is still at the level of recruiting volunteers for its Phase I.

Two Japanese pharmaceutical companies with specialty in developing vaccines are striving to enter the COVID-19 vaccines marathon. TAK-919 vaccine developed by Takeda uses the same formulation as the Moderna vaccine (mRNA-1273). The vaccine was registered for phase I/II on Healthy Japanese adults aged 20 years and older given two doses of the vaccine 28 days apart. DS-5670 vaccine developed by Daiichi Sankyo, Inc. The phase I/II trial is being conducted in Japan in a total of 152 healthy adults including elderly individuals aged 20–72 years to evaluate the safety and immunogenicity of the vaccine and thereby estimate the optimal dosage of DS-5670.

### 6.2. Influenza

Influenza virus infection poses many public health threats around the world that traditional vaccines failed to seize its continuous fatal outbreaks. Thus, hopes of RNA vaccines as being a savior led tremendous efforts to be invested in crafting potent vaccine for the great diversity of influenza virus strains. Their capability to elicit robust, protective immune responses against various pathogens has shed the light on their adoption to tackle influenza virus in both preclinical and clinical phases. Influenza mRNA vaccines, either self-amplifying or nonreplicating, have recently demonstrated adequate protection and promising efficacy in preclinical models [69]. On the basis of supporting preclinical data, two phase I clinical trials for H10N8 and H7N9 influenza virus mRNA vaccines using nucleoside-modified mRNA-LNPs encoding full-length H10 and H7 HAs were implemented to evaluate the immunogenicity and safety of mRNA influenza vaccines in humans [70,71]. Bahl and his colleagues reported interim findings for 23 vaccinated individuals who received 100 μg of H10N8 mRNA-LNPs vaccine intramuscularly, and then immunogenicity was measured 43 days after vaccination showed results suggesting that the vaccines were moderately immunogenic [70]. On the other hand, H7N9 candidate vaccine showed rapid immune response that was observed in participants with undetectable hemagglutination inhibition (HI) titers 43 days after the first 10 µg dose, showing robust antibody maturation. In addition, HI titers persisted for about 6 months postvaccination, resulting in developing memory B-cell responses. Safety and reactogenicity profiles for doses up to 100 µg of H10N8 and H7N9 mRNA vaccines appeared to be comparable to that of more traditional adjuvanted and unadjuvanted influenza vaccines [71].

### 6.3. Mosquito-Borne Diseases

Zika virus (ZIKV) infection is a disease of global health concern. Around eighty-six countries worldwide have reported the spreading of mosquito-transmitted zika infection in their territories, yet no vaccines are currently available for clinical intervention, making the development of zika vaccines a priority. mRNA vaccines encapsulated in lipid nanoparticles targeting the premembrane and envelope (prM-E) surface glycoproteins of ZIKV were rapidly developed inducing very high levels of neutralizing antibodies that demonstrates protective efficacy and complete protection in animal studies against challenge after a single intradermal dose or after prime and boost intramuscular immunization [72,73]. The first mRNA vaccine to target ZIKV, mRNA-1325, is a nucleoside-modified mRNA vaccine developed by Moderna. In December 2016, mRNA-1325 entered its phase I clinical trial to evaluate its safety and immunogenicity in healthy adults in a nonendemic zika region. Significant challenges facing the progression of ZIKV in phase III efficacy trials are the declining rates of ZIKV transmission, unpredictability of ZIKV outbreaks, the need for inclusion of vulnerable target populations as pregnant women and to the broad spectrum of clinical manifestations making a single definite endpoint difficult [74].

Same mRNA technology platforms used to develop zika vaccines have also been embraced in a battle against another mosquito-transmitted viral disease and chikungunya disease. Although a huge mass of people suffers from chikungunya endemic, the low funding and lack of awareness of the disease hindered the development of chikungunya vaccine (CHIKV) candidates, and currently, there are not any licensed vaccines to prevent chikungunya disease. Given that CHIKV antigen variety is limited and infection may lead to lifelong immunity, a design based on mRNA vaccine made the best use of this merit. By introducing the sequences encoding monoclonal antibodies (mAbs) into lipid-encapsulated mRNA, the candidate vaccine (CHKV-24) succeeded in inducing human IgG in mice and macaques animal models, which peaked at 24 h after immunization [75]. Another strategy was utilized to trigger immune response through expressing CHIKV structural polyprotein viral antigens. With just a single dose of this mRNA vaccine, nonhuman primates showed strong immune response and mice were 100% protected from CHIKV infection [76]. As a consequence of these promising results, Moderna’s CHIKV vaccine (mRNA-1388) was advanced for phase I study in humans which showed that mRNA-1388 vaccine was well-tolerated at the various doses administered (25, 50 or 100 µg). Neutralizing antibody titers increased in all study subjects in a dose-dependent manner with the 100 µg dose of mRNA-1388 accounting for the highest seroconversion followed with a substantial boost after the second dose and an associated 100% seroconversion in all subjects [77].

### 6.4. HIV

Human immunodeficiency virus (HIV), that attacks cells which help the body fight infection, making people more vulnerable to other infections and diseases, still does not have an efficient vaccine yet. The fast rates by which the virus mutates is challenging any advancements in HIV vaccine development. There can be a wide spectrum of HIV viral strains circulating in a single individual and within the population, each having a different genetic makeup, thus a robust HIV vaccine would have to be able to convey protection against many virus strains. Currently, there are several phase I and II clinical trials with mRNA vaccines against HIV. Several strategies were adopted for combating HIV using mRNA vaccines, starting with the Gandhi et al. study that reported unsatisfactory results of a clinical trial for immunization of HIV-1-positive participants using autologous dendritic cells (DCs) transfected with mRNA encoding HIV-1 structural proteins Gag and Nef and pulsed with keyhole limpet hemocyanin (KLH). Weak immune responses were observed, shedding light on DC vaccination improvement [78]. However, another substudy that adopted the same strategy developed AGS-004 vaccine that was based on matured autologous dendritic cells co-electroporated with in vitro transcribed RNA encoding autologous HIV antigens. Luckily, this vaccine induced a positive immune response when administered to participants with acute HIV infection and successfully ≥2-fold increase in antibody titers as well as induction of specific T-lymphocytes (CTLs) in all study participants [79]. A different strategy using a combination of HIV immunogen, known as HIVACAT T-cell immunogen (HTI), activation adjuvant TriMix and selected mRNA comprising 16 conservative fragments from HIV-1 structural proteins—Gag, Pol, Vif, and Nef—is a new mRNA-based therapeutic vaccine candidate against HIV-1. It showed good safety, tolerance and encoded a potent HIV recombinant antigen in preclinical models; however, phase II human trials reported an unexpected start codon that was found upstream of the HTI recombinant antigen coding sequence which likely had a negative influence on HTI protein expression [80]. Thus, it was impossible to draw any conclusions on the induction of cellular immune responses against the HTI immunogen [81]. This led us to consider choosing proper antigens and delivery systems that can trigger antigen-specific T-cell immune response should be emphasized at HIV mRNA vaccine design in the future.

### 6.5. Cytomegalovirus (CMV)

Currently, there are no approved vaccines for CMV, which is the most frequent cause of viral disease in transplant recipients resulting in transplant failure as well as the leading cause of disabling infections in newborns. mRNA-1647 was the first mRNA vaccine candidate for an infectious disease to enter a phase II clinical trial prior to the COVID-19 vaccine candidates. mRNA-1647 is based on six mRNAs encoding two antigens in one vaccine. These antigens are subunits of the CMV pentamer complex and the glycoprotein B (gB) protein which are both highly immunogenic and account for the first step in CMV infection which is entering the epithelial cells. Prior attempts only produced a single protein gB antigen, but this left the cells that are unlocked by the pentamer unprotected. The main problem was trying to make such a complex protein outside of the body in a way that could be used for large-scale production. Moderna encapsulated mRNA-1647 in a nanoparticle delivery system overcame this challenge as the antigen was successfully produced in vivo in preclinical models of mice and nonhuman primates. The mRNA-1647 vaccine can produce both the gB and pentameter proteins eliciting high levels of neutralizing antibodies and strong T-cell responses. Increases in antibody titers against both antigens were observed with increasing dose levels, which were boosted after a second or third dose of vaccine [82].

### 6.6. Rabies

Rabies mRNA vaccine was developed by CureVac after a long period of extensive study and development came to the light in the form of a sequence-optimized, chemically unmodified mRNA that encodes the rabies virus glycoprotein. CureVac’s CV7202 showed enhanced immunogenicity in mice and nonhuman primates that resulted from the LNP platform in addition to protamine complexed mRNA. This immunogenicity was supported with the activation of T-cell responses as well as the presence of IL-6 and TNF (tumor necrosis factor) in the draining lymph nodes and injection sites indicating positive immune response [83]. A phase I clinical trial was initiated, and a report on CV7202 concluded that the administration of CV7202 was generally safe, reasonably tolerable and elicited rabies neutralizing antibody responses after 2 doses that met WHO criteria in all study subjects [84]. 

### 6.7. Ebola Virus (EBOV)

Ebola virus is one of the most fatal viral infections worldwide. After the 2014–2016 epidemic of Ebola in West Africa, the WHO rushed the clinical trials and approvals processes for Ebola vaccines. The most well-known Ebola vaccine is the rVSV-ZEBOV vaccine which is a vesicular stomatitis virus (VSV) and expresses the EBOV glycoprotein (GP) [85]. While a durable antibody response is produced as a result of the vaccination, several studies have attempted to address the challenges faced by this vaccine through the design of alternative mRNA vaccines. A dendrimer-RNA nanoparticle showed promising results and elicited both CD8+ T cell and antibody responses; however, the large size of the amplicon is expected to create many challenges in the scale-up of the production of this vaccine [86]. Furthermore, a lipid nanoparticle encapsulated modified mRNA vaccine encoding the EBOV GP in a membrane-bound form showed promising results in animal model [87].

## 7. Challenges Faced by RNA Vaccination Technologies

### 7.1. Safety and Tolerability

Although the rapid pace of RNA vaccine development raised some level of hesitation against RNA vaccines, these vaccines surpass other traditional vaccines in having the potential to be much safer as they are considered noninfectious platforms that lack the viral structure, and the replicon does not produce infectious viral particles. Additionally, RNAs are also nonintegrating, and they do not integrate into the host genome and are degraded during the process of antigen expression. The main concern about safety of RNA vaccines is the possibility that these vaccines may generate strong type I interferon and proinflammatory cytokines responses that can promote the development of autoreactive B cells and T cells, posing an even greater threat which could lead to inflammation and autoimmune conditions [88,89]. One study reported the safety of mRNA vaccines among pregnant women who did not show obvious safety signals after receiving COVID-19 mRNA vaccines and recommended more longitudinal follow-up in order to identify maternal, pregnancy and infant outcomes [90]. Moreover, several studies tackled the safety and reactogenicity of SARS-CoV-2 mRNA vaccines in organ transplant recipients since immunosuppressed patients were excluded from original vaccine trials and reported no major safety concerns. In addition, symptoms were consistent with vaccine reactogenicity demonstrated in original clinical trials in healthy adults and those with stable, chronic medical condition [91]. Although two doses of mRNA vaccines elicited considerable immune response in organ recipients considering limited protection is better than none, a study suggests that many transplant recipients may remain at risk for COVID-19 after two doses of mRNA vaccine [92]. To overcome this issue, recommendation for a booster dose of mRNA vaccine to be administered to organ transplant recipients needs to be considered for better protection of those immunocompromised patients [93,94]. Data still need to tackle other safety aspects bearing in mind vulnerable populations, including children, elderly and patients with chronic conditions such as autoimmune disorders. Furthermore, RNA vaccine compatibility with different medical drugs also needs thorough evaluation. Thus, active and sentinel surveillance became a must to meticulously monitor and assess the safety profile of the vaccines. 

### 7.2. Immunogenicity

Most mRNA vaccine candidates require two rounds of injections to be effective. Questions regarding whether additional booster doses of RNA vaccines would be required for population, and if so, what would be the timing and dosage? To answer these questions, Moderna initiated phase I clinical trial to evaluate COVID-19 booster vaccine candidates: mRNA-1273.351 encodes the prefusion stabilized spike protein of the SARS-CoV-2 variant B.1.351 which was first identified in the Republic of South Africa and mRNA-1273.211, a multivalent candidate that combines mRNA-1273 ancestral strains and mRNA-1273.351 in a single vaccine [95]. Further research is needed to determine whether shots will be required over the year to maintain immunity or to be given annually like the flu shot.

### 7.3. Efficacy and Protection

The long-term efficacy and possible side effects of RNA vaccines are still obscure. A vaccine is considered efficient when it generates desired humoral and cellular immunity against the pathogen, besides minimizing adverse events. mRNA vaccine efficacy against COVID-19 was addressed in a study for the United States Centers for Disease Control and Prevention (CDC) that involved the two authorized mRNA vaccines for COVID-19: mRNA-1273 and BNT162b2. The study showed promising effectiveness of partial or full vaccination among hospitalized adults aged ≥65 years who are at higher risk of the disease [29]. The adjusted vaccine effectiveness (VE) was estimated to be 94% for full vaccination and 64% for partial vaccination, which corresponds with the efficacy on the same subgroup in clinical trials [29]. These findings are also consistent with the study that addressed the real-world effectiveness of SARS-CoV-2 vaccination by BNT162b2 including older adults [55]. On the other hand, variants of concern (VOCs) may reduce vaccine effectiveness, which may be evident by a high number of vaccine breakthrough cases or a very low vaccine-induced protection against severe disease. Currently, enhanced genomic surveillance in some countries has detected six variants of SARS-CoV-2 circulating; B.1.1.7 (first detected in the United Kingdom), B.1.351, the P.1 (first detected in Brazil), B.1.526 and B.1.525 (first detected in New York), B.1.427 and B.1.429 (first detected in California) and the B.1.617 variant that recently emerged in India. A study conducted by Goel et al. showed that mRNA-1273 and BNT162b2 mRNA vaccines elicit neutralizing titers against the B.1.351 South African variant that skyrocketed after the first dose in recovered subjects [96]. However, another study tested pseudovirus bearing the B.1.1.7 lineage spike protein with sera of study participants who were previously vaccinated with BNT162b2 showed a sixfold reduction of neutralization for the majority of sera yet preserved neutralizing titers against the B.1.1.7 lineage pseudovirus [97]. Despite being a highly contagious variant that is dominating nationwide, mRNA vaccines showed to offer protection and sustained effectiveness against the B.1.617.2 variant as well. A study held in Scotland showed that Pfizer-BioNTech mRNA vaccine demonstrated 79% effectiveness against COVID-19 after 14 days from receiving the second dose [98]. These results are consistent with the data published by Public Health England that reported 88% effectiveness after two doses of BNT162b2 [99]. Although mRNA vaccines are still efficient against the evolving circulating VOCs till this date, these variants still pose further concerns on whether mRNA vaccines will still be efficient in combating these rapidly mutating variants in the future or not. 

## 8. Storage and Stability

One of the outstanding challenges is in terms of chemical stability of the mRNA/excipients, mRNA is a large molecule with poor stability and the origin, quality, and supplier of mRNA vaccine excipients, as well as the design of the formulation manufacturing processes, can modulate the pharmaceutical stability of formulated mRNA vaccine candidates. Some mRNA vaccines formulas may require specific storage conditions such as the need for an ultra-cold storage or to be kept cold, which may be hard during the large-scale production and distribution of the vaccine [100,101,102,103].

These challenges make RNA vaccination prone to failure, especially in poor rural areas of tropical countries. Pfizer/BioNTech’s BNT162b2 requires storage and shipping at ultralow temperature between −80 and −60 °C (−112 to −76 °F), whereas Moderna’s mRNA-1273 requires temperature between 25 and −15 °C (−13 to 5 °F). In order to overcome this hurdle, Pfizer recommended alternative temperature for transportation and storage which received the FDA applaud to these recommendations as they allow Pfizer-BioNTech COVID-19 undiluted frozen vaccine vials to be transported and stored at conventional temperatures similar to that found in pharmaceutical freezers lasting up to two weeks which is considered more than enough for shipping the vaccine from one country to another [104]. In addition, Pfizer reported developing special shipping containers to meet potential logistical challenges to create equity in distributing the vaccine. Therefore, the development of thermostable mRNA vaccines is an urgent need and efforts should be invested in optimizing formulations of synthetic mRNA vaccines as they have shown the possibility to generate thermostable vaccines [27].

## 9. Conclusions and Future Perspectives

RNA vaccines have recently caught the scientific and public attention attracting massive academic and industrial investment. Thanks to biotechnology startups whose innovation and dedication brought us the outcome of years and years of extensive research in the form of an RNA vaccine shots paving the way to develop a wider innovative profile of another RNA vaccines. Even though some RNA vaccines have been approved, we do not know the long-term safety and efficacy of this new technology. Technically, mRNA vaccine development is often times hindered by a plethora of challenges starting from their large size, intrinsic instability and vulnerability to enzymatic degradation in addition to strict temperature requirements to maintain stability. Moreover, logistical and policy dilemmas: affordability, fair distribution in various countries, priority of professional individuals, dosage, vaccine hesitancy, repeat doses, and prohibitive costs stand as provocateurs for these vaccines to find its way smoothly to the public. The boost provided by the COVID-19 pandemic accelerating research and development of RNA vaccination should be strongly utilized in exploring novel strategies to tackle said challenges. These strategies should include the design of improved vectors and delivery systems. Enhanced delivery systems in particular have the potential to increase construct stability, cell targeting and translational efficiency, which are extremely poor in cases of naked mRNA. While lipoplexes and lipid-based nanoparticles have shown the most promise as delivery methods, it is also worth exploring polymers and lipid–polymer hybrid nanoparticles. Both strategies can provide great promise in terms of safety, stability, high transfection efficiency and low price. Continuous advancement in the field of RNA vaccination is direly needed as the technique holds promise of treatment and prevention of both communicable diseases and noncommunicable ones such as cancer.

## Figures and Tables

**Table 1:** Lipid-based formulations for nucleic acid delivery [33].

Traditional Liposomes	Used as drug carriers, biodegradable, which enhances efficacy and minimizes toxicity
Lipoplexes	Excluded from clinical trials due to poor encapsulation and tolerability
Cationic nano-emulsions (CNE)	Bind to self-amplifying mRNA, strong immune response with high levels of neutralizing antibodies and induction of T cell
Nanostructured lipid carriers (NLC)	Used for mRNA vaccine. Provides enhanced stability depending on amount of solid lipid used

**Table 2:** Composition of lipid nanoparticles (LNP) [33].

Ionizable Cationic Lipids	Strong Encapsulation. Environment pH Affects Its Charge Which in Turn Affects Fusion and Release of Nucleic Acid into Cytosol	
Helper lipids (PEG, cholesterol, and phosphatidylcholines)	Aids with the stability of NP, promoting uptake and delivery of the nucleic acid	
PEG Lipids	Cholesterol	Phosphatidylcholines
Controlling its amount is crucial since it affects the binding of LNP, thus affecting its accumulation or elimination in the blood.	Presence helps with stability, integrity in structure and LNP fusion	Helps in development and disruption of lipid bilayer aiding in the escape of endosome

**Table 3:** Summary of clinical studies assessing infectious disease mRNA vaccines.

Target	Vaccine Name	Developers	Trial Characteristics	Immunogenicity	Trial No.
SARS-CoV-2	mRNA-1273	ModernaTX, Inc. + National Institute of Allergy and Infectious Diseases (NIAID)	2 doses (0, 28 d) (100 μg) IM 18–55, 56+ years	Nab and CD4+ T-cell responses were observed in all participants with persistence lasting up to 3 months.	NCT04283461 (I) NCT04405076 (II) NCT04470427 (III) NCT04760132 (IV)
	mRNA-1273.351	ModernaTX, Inc. + NIAID	2 doses (0, 28 d) (25 μg, 50 μg, 100 μg) IM 18–99 years	N/A	NCT04785144
	BNT162b2	Pfizer/BioNTech + Fosun Pharma	2 doses (0, 21 d) (30 μg) IM 18–85 years	Increased RBD-binding IgG, NAb titers, CD4+ and CD8+ T-cell responses after a second dose. Immunogenicity persisted over a median of 2 months.	NCT04760132 (I) NCT04380701 (I/II) NCT04368728 (II/III) NCT04760132 (IV)
	CVnCoV	CureVac AG	2 doses (0, 28 d) (12 μg) IM 18+ years	Neutralizing antibody titers in participants after two injections were comparable to those of convalescent human sera.	NCT04449276 (I) NCT04515147 (II) NCT04652102 (II/III) NCT04674189(III)
	ARCT-021	Arcturus Therapeutics	2 doses (0, 28 d) (2 μg) IM 21–80 years	Favorable immunogenicity results for both single-dose and prime-boost regimens.	NCT04480957 (I/II) NCT04668339 (II)
	LNP-nCoVsaRNA	Imperial College London	(0.1 µg, 0.3 µg and 1 µg) IM 18–45 years	N/A	ISRCTN17072692
	ARCoV	Academy of Military Science (AMS), Walvax Biotechnology and Suzhou Abogen Biosciences	2 doses (0, 14 d/ 0, 28 d) (5 µg, 10 µg, 15 µg) IM 18–59 years	N/A	ChiCTR2000034112 (I) ChiCTR2100041855 (II)
	ChulaCov19	Chulalongkorn University	2 doses (0, 21 d) (10 µg, 25 µg, 50 µg, 100 µg) IM 18–55, 65–75 years	N/A	NCT04566276
	PTX-COVID19-B	Providence Therapeutics	2 doses (0, 28 d) (16 μg, 40 μg, 100 μg) IM 18–64 years	N/A	NCT04765436
		GlaxoSmithKline	2 doses (0, 1 month) (1 µg, 3 µg, 10 µg, 30 µg) IM 18–50 years	N/A	NCT04758962
	MRT5500	Sanofi Pasteur and Translate Bio	1 dose/2 doses (0, 21 d) (15 µg, 45 µg or 135 µg) IM 18–49, 50+ years	N/A	NCT04798027
	DS-5670a	Daiichi Sankyo Co., Ltd.	2 doses (10 µg, 30 µg, 60 µg, 100 µg) IM 20–72 years	N/A	NCT04821674
	TAK-919	Takeda	2 doses (0, 28 d) (0.5 mL) IM 20+ years	N/A	NCT04677660
Influenza H7N9 virus	VAL-339851	ModernaTX, Inc.	2 doses (0, 6 months) (10 µg, 25 µg, 50 µg) IM 18–49 years	Induced humoral immune responses and high seroconversion rates.	NCT03345043
Influenza H10N8 virus	VAL-506440	ModernaTX, Inc.	2 doses (0, 21 d) IM (25 µg, 50 µg, 75 µg, 100 µg, 400 µg) ID (25 µg, 50 µg) 18–64 years	Induced robust humoral immune responses and high seroconversion rates.	NCT03076385
Zika	mRNA-1325	ModernaTX, Inc. and Biomedical Advanced Research and Development Authority	2 doses (0, 6 months) (10 µg, 25 µg, 100 µg) IM 18–49 years	N/A	NCT03014089
Rabies	CV7202	CureVac AG	2 doses (0, 28 d) (1μg, 2μg) 1 dose (5 μg) IM 18–40 years	Induction of NAb responses. No cell-mediated immune responses detected after two shots of 1 and 2 μg dosages.	NCT03713086
Cytomegalovirus	mRNA-1647	ModernaTX, Inc.	3 doses (0, 2, 6-month) IM 18–40 years	N/A	NCT04232280
HIV-1	AGS-004	Argos Therapeutics+ McGill University Health Centre+ Université de Montréal	4 doses (4-weeks apart) ID 18–65 years	AGS-004 dendritic cell administration increased multifunctional HIV-specific CD28+/CD45RA − CD8+ memory T-cell responses in all participants.	NCT00381212 (I) NCT01069809 (II)
	iHIVARNA-01	Erasmus Medical Center	3 doses (2-weeks apart) Intranodal 18+ years	Interim analysis did not show sufficient immunogenicity of patients compared to placebo.	NCT02413645 (I) NCT02888756 (II)
		Massachusetts General Hospital	4 doses (weeks 0, 2, 6, and 10) ID 18–65 years	Study participants developed de novo CD4 and CD8 proliferative responses to KLH and CD4 proliferative responses to Nef that were short-lived.	NCT00833781
Chikungunya	mRNA-1388	ModernaTX, Inc.	2 doses (0,28 d) (25 μg, 50 μg, 100 μg) IM 18–49 years	NAb titers increased significantly and boosted after the second vaccination.	NCT03325075

## Data Availability

Not applicable.
